# That Woodbridge Treatment for Typhoid Fever

**Published:** 1898-07

**Authors:** 


That Woodbridge Treatment for Typhoid Fever.—One of its virtues is, that the doctor must get in his work early, and keep it up; must give "No. 1" every "fifteen minutes" for two or three days (and nights). Now if that is'nt just enough to kill a Sampson—enough to Schley the stoutest man, let alone a poor fellow in the grip of typhoid, flat on his back and can't help himself. It is the worst piece of absurdity that could be perpetrated, to wake a patient every fifteen minutes, or rather, to keep him awake, to give him medicine. Some of you Texas doctors: you be the patient and let some fellow come at you a la Woodbridge. You wouldn't *do a thing* to that doctor but put a head on him—if you survived the treatment, and I think if it were I, I should make a big effort to do it before I got up. Think of lying awake looking at the clock and expecting "every fifteen minutes" to be called on to swallow No. 1, and every half hour to swallow No. 2 and every hour, No. 3. A real good and sympathetic nurse would have too much regard for your comfort to let the doses all fall on one hour, to give you No. 1, No. 2 and No. 3 all at one time; he would see that you got No. 3 at 2, No. 2 at 7½ minutes after 2, and consequently No. 1 at 5 minutes, and twenty minutes after, and again at 35 past, and then at 3. No, that would make it collide with No. 2. Oh pshaw! It would require a pretty smart nurse to keep the doses separate. Doctor, speaking of *don'ts*, don't try the Woodbridge on any body; it would be taking an unfair advantage of a patient, and if he survives he may lay for you to get even. *Don't.*

